# Jejunal Adenocarcinoma: A Rare Cause of Small Bowel Obstruction

**DOI:** 10.7759/cureus.21195

**Published:** 2022-01-13

**Authors:** Jay Patel, Hao Zhang, Chaudhry Saad Sohail, Matthew Montanarella, Mujtaba Butt

**Affiliations:** 1 Internal Medicine, Orange Park Medical Center, Orange Park, USA; 2 Gastroenterology, Borland Groover Clinic, Jacksonville, USA

**Keywords:** gi tumor, small bowel obstruction, small bowel malignancy, jejunal mass, jejunal adenocarcinoma

## Abstract

Jejunal adenocarcinoma (JA) is both a rare type of gastrointestinal malignancy and an uncommon cause of small bowel obstruction (SBO). It typically presents with vague symptoms, such as abdominal pain, nausea, vomiting, and, in some cases, weight loss. Due to this vague presentation as well as lack of definitive imaging techniques, diagnosis tends to be delayed and patients typically present at later stages. We present a case of a patient who presented with acute onset abdominal pain. Imaging revealed the presence of an SBO with the presence of a suspicious small bowel stricture. He eventually underwent upper endoscopy to find the mass, with subsequent biopsy indicating JA. We hope to bring greater awareness to jejunal carcinoma as a potent cause of SBO in adults.

## Introduction

Abdominal pain is a common symptom of patients presenting to the emergency room and encompasses a wide differential. Most commonly caused by adhesions (60%), small bowel obstruction (SBO) often presents with abdominal pain and frequently occurs in patients who have undergone prior abdominal surgical procedures. Other less frequent causes include hernias, Crohn’s disease, and volvulus [1]. Bowel obstructions caused by cancer are referred to as malignant bowel obstructions. They are most commonly caused by malignancy of the colon (25-40%), ovary (16%-29%), and stomach (6%-19%), with the pancreas and bladder being less common. They are the presenting symptom of advanced cancer in only 2% of cases [2]. SBO can rarely be caused by a small bowel adenocarcinoma (SBA). These encompass only around 4% of gastrointestinal (GI) malignancies, with duodenal adenocarcinoma (57%) composing the majority of cases and jejunal only around one-third (29%) [3,4]. Jejunal adenocarcinomas (JAs) represent a very uncommon subset of GI malignancies and therefore are a very rare cause of SBO in adults.

JAs occur most commonly around the age of 60 and present with vague, non-specific symptoms such as abdominal pain, nausea, and vomiting. In some cases, they are often diagnosed emergently as bleeding or obstruction. The vague presentation, underappreciated prevalence, and poorly defined diagnostic modalities can lead to its delayed diagnosis, overall potentially leading to a poorer prognosis. Some reports indicate over 80% of JAs being diagnosed at stage III or stage IV disease [5]. It is estimated that only 50% of patients can be cured with five-year survival rates at 67.5% [6]. Greater awareness of JAs is needed to enhance diagnostic success and improve prognosis.

## Case presentation

Our patient is a 59-year-old African American male with only relevant past medical history of uncontrolled hypertension who presented to the emergency department endorsing severe abdominal pain associated with intractable nausea and vomiting. According to the patient, his symptoms started four days prior to presentation, and there were no similar episodes in the past. Despite attempts at diet modification, his symptoms persisted, prompting his visit.

**Figure 1:**
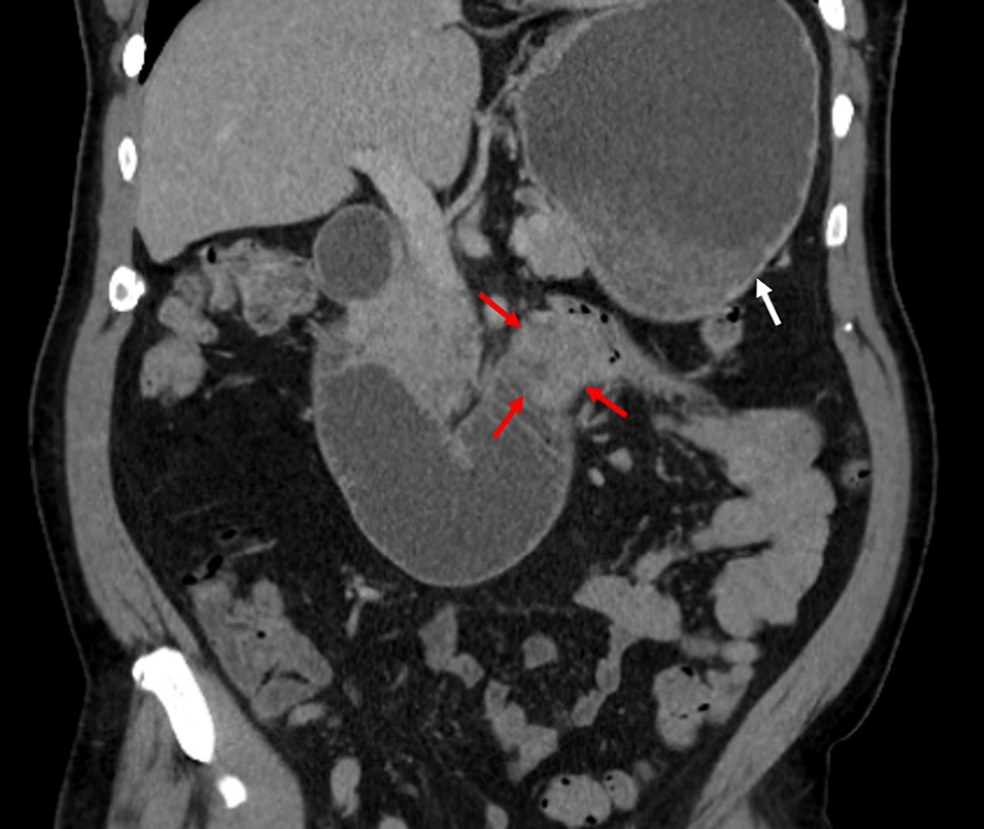
Contrast-enhanced CT scan of the abdomen demonstrating a soft tissue density at the duodenojejunal junction (red arrows) and a dilated stomach (white arrow).

Initial workup and blood work showed hypertensive emergency with acute kidney injury. The patient was eventually started on nicardipine drip to manage his blood pressure. Computerized tomography (CT) scan of the abdomen/pelvis without contrast was performed, which demonstrated a soft tissue density at the duodenojejunal junction measuring 3.2 x 2.0cm (Figure 1).

**Figure 2:**
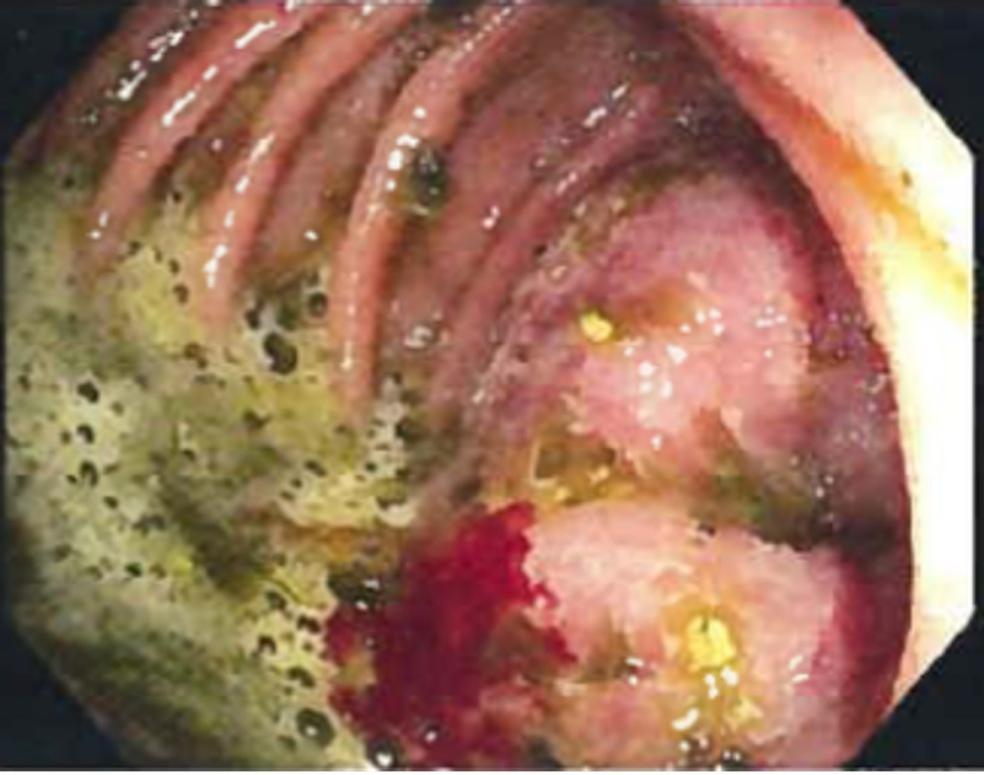
Intraoperative endoscopic image depicting a large frond-like/villous mass.

**Figure 3:**
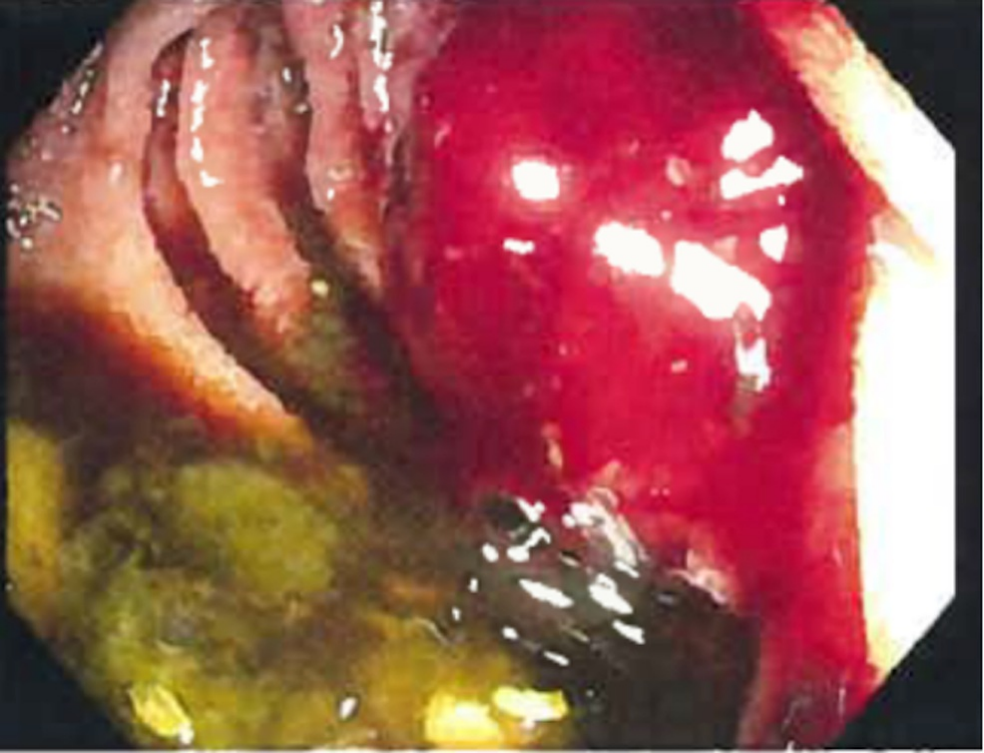
Intraoperative endoscopic image depicting a large frond-like/villous mass.

The patient was decompressed with nasogastric suction and scheduled for upper endoscopy. Endoscopy showed an exophytic mass at the level of the jejunum causing partial SBO (Figures 2, 3).

**Figure 4:**
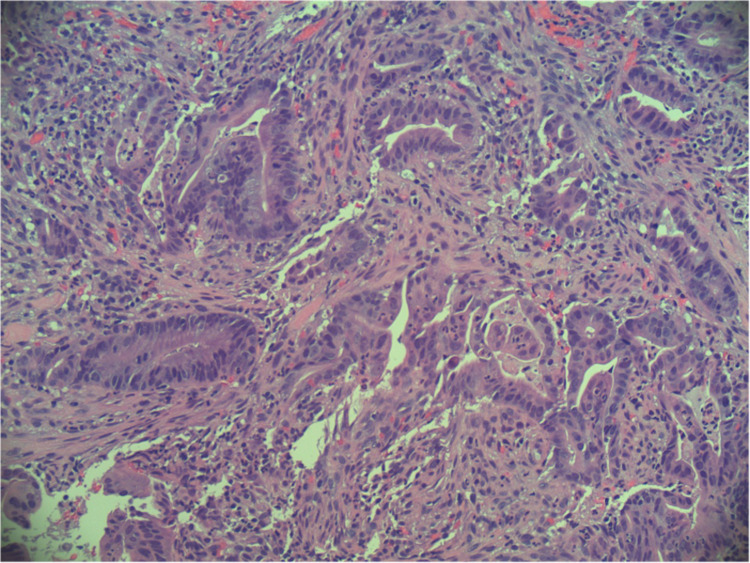
Hematoxylin-eosin stain at 20x magnification: tumor fragment with disordered, complex glands in a desmoplastic stroma.

**Figure 5:**
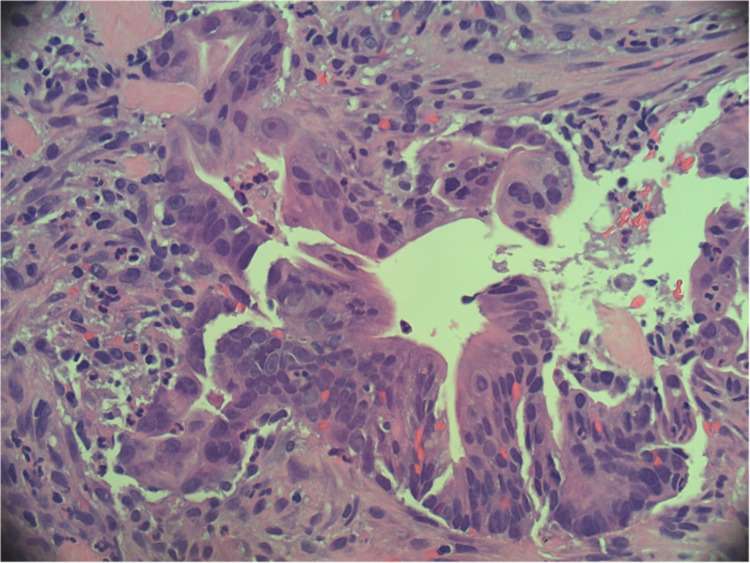
Hematoxylin-eosin stain at 40x magnification: tumor fragment with cellular atypia and prominent nucleoli.

The area was successfully injected with India ink for tattooing. Biopsy of the mass demonstrated the architectural and cellular features of jejunum adenocarcinoma, and a diagnosis was made (Figures 4, 5).

The patient was scheduled for surgical resection; however, the mass was deemed unresectable due to its proximity to the superior mesenteric artery. He finally underwent gastrojejunal bypass. The patient then followed up as an outpatient for cancer marker identification and targeted chemotherapy.

## Discussion

Despite the small bowel encompassing around three-fourths the complete length of the GI tract, SBAs represent some of the rarest of GI malignancies, with rates as low as 1 case per 100,000 individuals, and reported to be less than 5% of all GI malignancies [7,8]. A variety of hypotheses have been proposed to explain this phenomenon. The overall environment of the small bowel is seen as being anti-neoplastic, as potentially explained by the fast transit of food and higher IgA levels with more extensive lymphoid tissue. There is also a more rapid turnover of cells and lower bacterial count, as compared with the colon [4,9].

Risk factors for JA include alcohol use, smoking, preexisting adenoma, Crohn’s disease, Lynch syndrome, and Puetz-Jeghers syndrome among others, none of which our patient had [7]. Presentation often occurs at the age of 55-65 with vague symptoms of abdominal discomfort, nausea, vomiting, and potentially bleeding. Due to this non-specific presentation, diagnosis can often be delayed until advanced stages. Reports have indicated that 40% of patients present at stage III (lymph node metastasis) and around 40% present at stage IV (distant metastasis) [5].

CT scan remains the preliminary modality of choice for patients presenting with GI symptoms; however, studies depict only around a 50% accuracy in detecting lesions, lacking data regarding the intestinal mucosa and often missing smaller and flatter lesions [10]. Overall imaging diagnosis remains challenging, with a majority of cases properly diagnosed intraoperatively and subsequently confirmed via histopathological analysis [11]. Barium follow-through has a sensitivity up to 50% in the detection of small bowel malignancy [12]. Traditional endoscopic techniques are limited in their ability to full visualize the small bowel. Double balloon and push endoscopy overcome this by being able to extend to a more distal part of the intestinal tract but may be limited to more advanced centers [11]. Distal tumors may require the use of video capsule endoscopy or double balloon enteroscopy; however, with respective diagnostic yields of 20-30% and 60-70%, respectively, further testing may be needed. Baseline CEA and CA 19-9 assays are recommended for prognostic assessment [13].

The treatment of choice remains complete resection, with complete tumor removal with clear margins proposing the best overall survival [14]. Although studies have been proposed regarding the potential for chemotherapy in SBA, it does not appear to provide any significant effect on patient survival. Since most of these studies involved duodenal adenocarcinoma, it is difficult to comment on the use of chemotherapy in JA. Curative resection therefore offers the best promise with rates of cure up to 50% [11].

## Conclusions

Our case represents a small subset of patients with JA who may develop small bowel obstruction as a result of tumor invasion and growth. With the paucity of data on this subject in the medical literature, the mainstay of diagnosis involves biopsy and histopathological analysis. In our case, the patient was planned for surgical intervention; however, due to late presentation of the mass and spread to surrounding vasculature, it was deemed not resectable. This paper was written to shed light on the clinical diagnosis of small bowel malignancy and a unique presentation leading to diagnosis. At this point, little is known about the etiology of these small bowel malignancies. Future studies with larger sample populations and molecular diagnostic datasets could pave the way to establishing targetable tumor markers and sound treatment approaches. We feel that this process starts with a greater awareness of the pathology and its potentially unique presentations in an attempt to better aid patients affected with this GI malignancy.
